# Molecular Analysis of *Anaplasma phagocytophilum* Isolated from Patients with Febrile Diseases of Unknown Etiology in China

**DOI:** 10.1371/journal.pone.0057155

**Published:** 2013-02-22

**Authors:** Lijuan Zhang, Guiqiang Wang, Qinghui Liu, Chuangfu Chen, Jun Li, Bo Long, Hong Yu, Zhilun Zhang, Jing He, Zhangyi Qu, Jiguang Yu, Yuanni Liu, Tuo Dong, Na Yao, Yong Wang, Xueqin Cheng, Jianguo Xu

**Affiliations:** 1 Departments of Rickettsiology, National Institute for Communicable Disease Control and Prevention, Chinese Center for Disease Control and Prevention, Beijing, China; 2 Departments of Infectious Diseases, Peking University First Hospital, Beijing, China; 3 Departments of Intensive Care, Shandong Laizhou People's Hospital, Laizhou, Shandong Province, China; 4 Animal Science & Technology College, Shihezi University, Shihezi, Xinjiang Province, China; 5 Departments of Infectious Diseases, Yantai Infectious Diseases Hospital, Yantai, Shandong Province, China; 6 Departments of Epidemiology, Tianjin Center for Disease Control and Prevention, Tianjin, China; 7 Departments of Infectious Diseases, 302 Hospital of PLA, Beijing, China; 8 School of Public health, Harbin Medical University, Harbin, Heilongjiang Province, China; Auburn University, United States of America

## Abstract

Although anaplasmosis cases have been nationally identified in China, no human isolates of *A. phagocytophilum* have been obtained, which limits the analysis of any molecular and genetic contributions to patients' severe clinical manifestations and the study of the bacteria's pathogeneses in China. Given this situation, a joint project was conducted in 2009–2010. A total of 421 febrile cases of unknown etiology were collected and the patients' blood samples were collected for laboratory diagnoses including serologic diagnosis based on the four-fold rise in the anti- *A. phagocytophilum* IgG titer by indirect micro-immunofluorescence assay (IFA), positive PCR assay and confirmation of *A. phagocytophilum* DNA and positive culture of *A. phagocytophilum* and confirmed by amplification and sequencing of the 16S rRNA and *ank A* genes of the *A. phagocytophilum* isolates. A total of 570 ticks were collected from the patients' domestic animals (456) and from wild fields (114) for culturing and amplifying and sequencing the 16S rRNA gene of *A. phagocytophilum*. Phylogenetic analyses were performed on the 16S rRNA and *ank A* gene sequences of the isolates and the ticks tested in the study. A total of 46 (10.9%) confirmed and 16 (3.8%) probable cases were diagnosed and severe clinical features and higher mortality rates were observed in these Chinese patients. Five isolates were obtained and the 16S rRNA genes of the 5 isolates were conserved but variety for *ank A* genes. Two human isolates and 1 tick isolate from Shandong Peninsula, where all patients exhibited severe clinical manifestations, were grouped as one clan based on the phylogenetic analyses, while 2 other human isolates were clustered in a second clan. 43.5% of *H. longicornis* were infected with *A. phagocytophilum*.The present study is the first to obtain clinical isolates of *A. phagocytophilum* in China. The diversity of the *ank A* genes of Chinese isolates will help us to further discern the relationship between the variations in the *ank A* genes and the severity of the disease's clinical manifestations in China.

## Introduction

**Figure 1:**
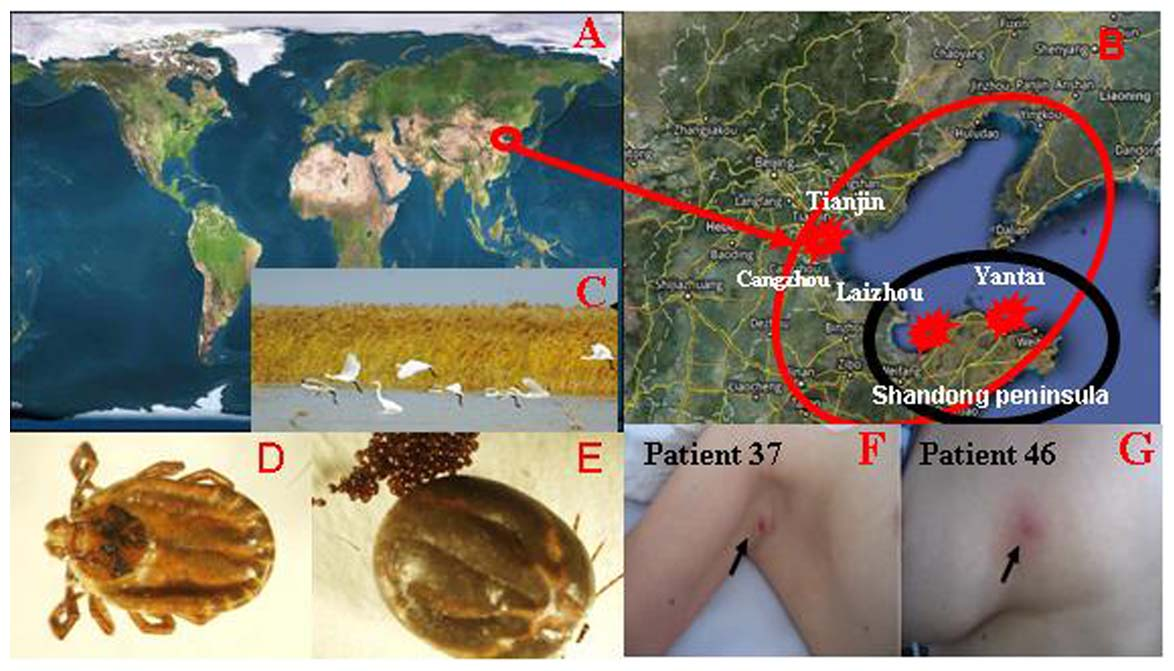
Map of China and the Bohai Sea Ring areas,Shandong Province. The Laizhou Bay, Shandong Province (B:areas enclosed by the black circle), where the 2 human isolates (LZ-HGA-agent-3 and LZ-HGA-agent-4) and the tick isolate (LZ- HGA-agent-T1) isolated. The *H. longicornis* collected in the Laizhou Bay (D and E). Images of HGA patients with eschars from the Laizhou Bay, Shandong Province (F and G).

Anaplasmoses are emerging tick-borne rickettsial diseases (TBRDs) caused by the obligate intracellular bacteria *Anaplasma phagocytophilum* [1], [2], [3]. In 2006, an unusual nosocomial human-to-human transmission of anaplasmosis occurred in Anhui Province, China [4]. An investigation of the seroepidemiological status of the zoonotic rickettsial infection caused by *A. phagocytophilum* in Tianjin City, one of the largest municipalities and the biggest trade port in the northern part of Bohai Bay (Figure 1 B), revealed that the average seroprevalence was 8.8% [5]. Subsequently, a broad investigation was conducted to assess the epidemiological status of *A. phagocytophilum* among farmers and domestic animals in 10 Provinces/Cities in China. The investigation showed that the average seroprevalence of *A. phagocytophilum* was 13.9% in farmers (unpublished data) and 10.1% in dogs, 3.8% in goats and 0.7% in cattle respectively [6]. Despite clear serological and molecular evidence demonstrating the existence of *A. phagocytophilum* infections in humans, domestic animals, ticks, and rodents in China [6], [7] and even etiologic evidence of infections in rodents [8], no positive cultures of the microorganism have been documented in clinical patients with anaplasmosis in China. Furthermore, a recent report indicated that Chinese patients have severe clinical symptoms and 45.8% of patients had systemic inflammatory response syndrome (SIRS), while 30.1% of patients had multiple organ dysfunction syndrome (MODS) [9]. Consequently, the isolation of Chinese *A. phagocytophilum* agents is very important for furthering our understanding of the molecular basis of human-to-human *A. phagocytophilum* transmission, further studying the pathogenesis of Chinese *A. phagocytophilum* isolates and developing rapid diagnosis reagents and a vaccine for the disease. A nationwide prospective investigation of clinical and microbiological characteristics of the emerging zoonotic rickettsial infection and related tick vectors was undertaken collaboratively by the Department of Rickettsiology, the National Institute for Communicable Disease Control and Prevention, the China CDC, five clinical hospitals (Peking University First Hospital, Laizhou People's Hospital in Shandong Province, 302 Military Hospital of the People's Republic of China, Yantai Infectious Diseases Hospital in Shandong Province and Beijing Friendship Hospital) and the Tianjin CDC between March 2009 and October 2010.

## Materials and Methods

### Case definitions and blood sampling

The research protocol was approved by the ethics committees of China CDC, and all the patients gave written informed consent before blood sampling. According to the diagnostic criteria proposed by US CDC [1], confirmed cases in the study were required to meet the following criteria: 1)Demonstration of 4-fold change in antibody titer to *A. phagocytophilum* antigen by IFA in paired serum samples; 2) Positive PCR assay and confirmation of *A. phagocytophilum* DNA; 3) Identification of morulae in leukocytes and a positive IFA titer to *A. phagocytophilum* antigen; 4) Culture of *A. phagocytophilum* from a clinical specimen. Probable case must meet the following criteria: 1) Identified in a person with a clinically compatible illness with a single positive IFA titer(IgM≥1∶80 or IgG≥1∶160) to *A. phagocytophilum* antigen;2) Visualization of morulae in leukocytes.

After receiving patient consent, 2 ml of non-anticoagulated blood was collected during the acute stage of illness, and the serum was separated to test for serological diagnosis. The remaining blood samples were used to extract DNA for molecular diagnosis. At the same time, 2 ml of EDTA-anticoagulated blood was collected for culture agents and to test whether *A. phagocytophilum* appeared in the neutrophils using Wright's stain. During the convalescent stage of illness, another 2 ml of non-anticoagulated blood was collected for serological diagnosis using a 4-fold change (i.e., a 4-fold increase or decrease) in IgG-specific antibodies. All of the samples were immediately transported to the Department of Rickettsiology, National Institute for Communicable Disease Control and Prevention, China CDC, within 12 h.

### Laboratory diagnosis

#### Serological assay

Paired sera from the acute and convalescence stages of illness were tested for the presence of IgM and IgG antibodies against *A. phagocytophilum* by immunofluorescence assays (IFAs) using the commercial FOCUS diagnostic reagents kit (REF: IF1403-A; Lot 102048, Cypress, CA, USA) according to the manufacturer's instructions. To differentiate *A. phagocytophilum* from other rickettsiae infections, the sera were also tested using IFA, as proposed by the WHO [10]. Ten common members of the order Rickettsiales were found, namely *R. prowazakii*, *R. typhi*, *R. heilongjiangensis*, *O. tsutsugamushi* types Karp and Kato, *C. burnetii*, *E. chaffeensis*, *R. felis*, *B. henselae* and *B. quintana*. Antigens were prepared by inoculating the above mentioned rickettsial strains in L929 or DH82 cells; the cultures were then collected when either the Gimenez or Wright staining revealed positive results, the cultures were ultrasonically crushed, and the bacteria were purified using density ultracentrifugation. A positive control serum was prepared by inoculating rabbits with the above mentioned standard rickettsiae strains.

#### Molecular assay

DNA was extracted from the blood samples from the acute stage of illness using a QIAamp Blood and Tissue kit (Qiagen, Hilden, Germany) according to the manufacturer's instructions. Sterile deionized water was used as a negative control. DNA from the *A. phagocytophilum* strain Webster, generously provided by JS Dumler at the Johns Hopkins University School of Medicine, USA, was used as a positive control. To enhance the sensitivity of the diagnosis, 3 types of molecular assays, namely nested PCR, real-time PCR and LAMP, were used in this study. The nested PCR targeting the 16S rRNA gene of *A. phagocytophilum* was performed as follows [11]: *Anaplasma* genus-specific outer-1 (5′-TTG AGA GTT TGA TCC TGG CTCAGA ACG-3′) and outer-2 (5′-CAC CTC TAC ACT AGG AATTCC GCT ATC-3′) primers were used in the first round of amplification, and species-specific HGA1 (5′-GTC GAACGG ATT ATT CTT TAT AGC TTG -3′) and HGA2 (5′-TATAGG TAC CGT CAT TAT CTT CCC TAC-3′) primers were used for the nested PCR. A final 389-bp DNA fragment was produced. In the first round, 10 µl of DNA was used for the templates, and 0.5 µl of the first-round PCR products was used as templates for the nested PCR. The bidirectional sequencing of positive PCR products was commercially conducted by Shanghai Shengong Biotechnology Co. (Shanghai, China). Briefly, the real-time PCR targeting the *msp2* gene was conducted as follows [12] : *Anaplasma* genus-specific MF (5′-GTA TTG GTG GTG CCA GGG TT-3′) and MR(5′-AGT GGT AGT AAG GAA GAT GAA GCT G-3′) primers and TaqMan MGB probes (FMA-TTA CGA GCG CTT CAA GAC-MGB) were designed and produced by Shanghai GeneCore BioTechnologies Co. Ltd. (Shanghai, China). The amplification conditions were as follows: 95°C predenaturation for 10 min followed by 45 cycles at 95°C for 40 sec and 60°C for 1 min. To test the samples, 10^8^–10^2^ copies/µl of recombinant plasmid solution was used for quantification. To eliminate false positives, each sample was tested in triplicate within the same run, and samples with a value of Ct ≤38 in a single test were considered positive. The LAMP assay primers were designed using PrimerExplorer V4 software (http://primerexplorer.jp) (Eiken Chemical Co. Ltd., Tokyo, Japan) and were based on conserved sequences determined by aligning 82 of the *msp2* gene sequences obtained from GenBank and synthesized by Sangon Biotech (Shanghai, China) [13]. The LAMP primer sequences were as follows: FIP(5′-TTGTIIGACTCIAGCTTTACACTCTGGGAGAGTAACGGAGAGA-3′) and BIP(5′-TGACTGGAACACICCTGATCCITIACCAACACTICCTTCC-3′); F3 (5′-CAGCGTTTAGCAAGATAAGAGA-3′) and B3 (5′-TCTCAAGCTCAACCCTGG-3′); and LF (5′-CCTTTAAGTATGGATATACTGCCTT-3′) and LB (5′-TTGGGTTTAAGGACAACATGCT-3′). The LAMP reactions were performed with the Loopamp Kit (Eiken Chemical Co. Ltd., Tokyo, Japan) in a 25-µl mixture containing 1.6 µM (each) FIP and BIP primers, 0.8 µM LF and LB primers, 0.2 µM F3 and B3 primers, 20 mM Tris-HCl (pH 8.8), 10 mM KCl, 8 mM MgSO_4_, 10 mM (NH_4_)_2_SO_4_, 0.1% Tween 20, 0.8 M betaine, 1.4 mM (each) deoxynucleoside triphosphates (dNTPs), and 1 µl *Bst* DNA polymerase (8 U/µl). The reaction mixture was incubated in an LA200 real-time turbidimeter (Teramecs, Tokyo, Japan) at 63°C for 60 min and then at 80°C for 5 min to terminate the reaction. The positive and negative samples were detected using electrophoresis on 2% agarose gels with ethidium bromide staining.

#### Morulae examination

The observation of the bodies of *A. phagocytophilum* in the neutrophils of patient blood samples provides important diagnostic clues. Briefly, blood smears were prepared as follows. First, 2 ml of EDTA-anticoagulated blood was centrifuged at 1500 rpm for 5 min. The plasma was then discarded, and 100 µl of fresh buffy coat was used to prepare smears centrifuged using a 7620 cytocentrifuge (WESCOR, Inc., Logan, UT, USA) according to the manufacturer's instructions. The prepared blood smears were stained using Wright's stain; specifically, 1.0 ml of Wright's stain was placed on the smear for 1 min. One milliliter of phosphate buffer, pH 6.5, was added for 5 min, and the stained smear was then rinsed with water. The film was allowed to dry, and the stained blood smears were then observed under a microscope.

#### Isolation and identification of bacteria

The blood samples of patients were inoculated into HL60 cells and cultivated as previously described [14]. The fresh buffy coats were prepared using the methods described in the “Morulae examination” section. Five hundred microliters of fresh buffy coat was inoculated into 15 ml of an HL60 cell suspension at 2×10^5^ cells/ml, and the cultures were incubated at 37°C in a 5% CO_2_ environment. For the tick samples, fully engorged ticks were disinfected in 75% alcohol for 30 min and then rinsed with sterile deionized water 3 times for 10 min each. The blood lymph from 10 to 20 feeding ticks was taken as one sample pool and centrifuged at 1500 rpm for 5 min to pellet the tissue fragments, and an aliquot (500 µl) of the suspension was inoculated into 15 ml of a HL60 cell suspension in the presence of penicillin (100 U/ml) and streptomycin (0.1 mg/ml). The culture was examined every 2 to 3 days using Wright's stain, as described in the “Morulae examination” section. Positive cultures were further confirmed by IFA using the specific IgG antibody against *A. phagocytophilum*. Briefly, the culture smears were prepared as described in the “Morulae examination” section. Rabbit anti-*A. phagocytophilium* IgG antibody (previously produced in our laboratory by inoculating rabbits with the *A. phagocytophilum* strain Webster) was added to the prepared culture smears, and the smears were incubated for 60 min in a moist chamber at 37°C. After washing to remove unbound antibody, the smears were reacted with fluorescein isothiocyanate-conjugated goat anti-rabbit IgG (Sigma Co.). The smears were rinsed again and then counterstained with Evan's blue before examination using a fluorescence microscope (Nikon, Tokyo, Japan). The culture smears were interpreted as positive when clear, fluorescent bodies were observed in the cytoplasm of HL60-cultured cells. Samples prepared with 3% nonfat powdered milk in PBS (pH 6.5) and normal rabbit serum were used as a negative control. DNA was extracted from the confirmed positive cultures for further molecular identification. The amplification of the 16S rRNA gene was performed using a pair of primers [15] (16S-FD1: 5′-AGA GTT TGA TCC TGG CTC AG-3′ and 16S-RP2: 5′-ACG GCT ACC TTG TTA CGA CTT-3′) at an annealing temperature of 56°C. In addition, a pair of primers targeting the *ank A* gene (PF, position from 679 nt to 697 nt: 5′-CGTATGGATCACCAGAAAG-3′; PR, position from 2464 nt to 2482 nt: 5′-CATTTGCTTCTTGAGGAGT-3′) were used to identify the isolates because they were specific and unique to *A. phagocytophilum* [16]. All PCR products were confirmed by commercial sequencing (Shanghai Shengong Biotechnology Co., Shanghai, China) using an ABI 3730 sequencing apparatus (Life Technologies, USA) and a Bigdye Terminator V3.1 sequencing kit. The sequences were compared with sequences available from the GenBank database.

### Tick collection and molecular assays

Based on the patients' histories of tick bites or tick exposure, we retrospectively went to the patients' hometowns to collect ticks from domestic animals, including cattle, dogs, goats and sheep. The ticks were kept alive in clean containers and then transported to the Department of Rickettsiology, National Institute for Communicable Disease Control and Prevention, China CDC, via the highway within 24 h. After classification, the ticks were disinfected using the same procedure described in the “Isolation and identification of bacteria” section. Subsequently, 5 to 10 partially engorged ticks or a single fully blood-engorged tick per sample pool were ground, and a total of 198 homogenized tick sample pools were prepared. DNA was extracted from the homogenized ticks, and the *A. phagocytophilum* 16S rRNA genes were amplified using the nested PCR method described in the “Molecular assay” section [11]. The PCR fragments were commercially sequenced and compared using the same procedure used to identify the isolates.

### Statistical analysis of clinical data

With the assistance of the Division of Health Statistics, National Center for Public Health Surveillance and Information Services, China CDC, the clinical data analysis was performed using SAS software (Version 9.1, SAS Institute, Inc., Cary, NC). Comparisons of the clinical data and analyses of the relationships among genders and areas were performed using the student's t-test and the chi-square test. Significance was defined as a P value of 0.05. The sequences were edited and assembled using the SeqMan program of the DNASTAR package (Lasergene, Madison, WI).

### Phylogenetic analysis

The phylogenetic analysis of the 16S rRNA and *ank A* genes of the isolates and tick samples was performed using the MEGA5.05 software, and the genetic tree was constructed using neighbor-joining (NJ) methods, with the complete deletion option, based on the Kimura 2-parameter model for nucleotide sequences. Bootstrap analysis was conducted with 1000 replicates. To construct the 16S rRNA phylogenetic tree, 14 sequences [17], [18], [19], [20], [21], [22], [23], [24], [25], [26], [27] from isolates in other parts of the world were included (Table 1). In addition, the sequence (EF211110), identified in a patient from the outbreak of nosocomial human-to-human transmission of anaplasmosis in Anhui Province in 2006 [4], and the sequence (EU982709), identified in a patient from Yiyuan County in 2007 [24], was analyzed. A partial sequence of the *Elusimicrobium* sp 16S rRNA gene (FM876310) was also included as a control sequence. For constructing the phylogenetic tree of *ank A*, the *ank A* sequences of the 5 isolates and another 10 sequences [18], [25], [27] from throughout the world were included (Table 1). The *ank A* sequence of the strain HZ was used as a reference sequence in the study.

**Table 1:** Sequences used in phylogenetic analysis based on the 16S rRNA gene and *ank A* gene of *A. Phagocytophilum*.

Gene	No. of GenBank accession	Name of isolates	Sources	Country	References
16S rRNA	EF211110	AH-HGA-1	Human	China	4
	AY969014	J4-3-6	*Ixodes ovatus*	Japan	17
	AY527214	Strong	horses	Sweden	
	CP000235	HZ	human	United States	18
	GU064899	HLAP327	*Haemaphysalis longicornis*	Jeju Island, South Korea	19
	GQ412337	China-C-Aa	*Apodemus agrarius*	China	20
	GQ412338	China-C-Y	sheep	China	20
	GQ412339	China-C-Tt	*Tscherskia triton*	China	20
	FJ968659	YN06-453	*Niviventer niviventer*	China	
	FJ968656	YN06-379	*Niviventer eha*	China	
	AB211164	*A. central*	Ticks	Japan	21
	AB211163	*A. bovis*	wild deer	Japan	21
	FJ389576	szg-3	Goat	Shizhu City, China	22
	FJ389577	wlg-3	Goat	Wulong City, China	22
	HQ629917	Rus30-13	*Ixodes ricinus*	Russia	23
	EU982709	YYH3	Human	China	24
*ankA*	GU236907	Roe deer 794	*Capreolus capreolus*	Slovenia	25
	GU236800	Human-1566	Human	Slovenia	25
	GU236863	horse-S3041-06	*Equus caballus*	Germany	25
	GU236851	dog-54	*Canis lupus*	Germany	25
	GU236795	SH-13	*Ovis aries*	Germany	25
	GU236719	red-deer-474	*Cervus elaphus*	Slovenia	25
	GU236744	bison-20	*Bison bonasus*	Poland	
	GU236806	human-03HE	Human	United States	25
	GU236807	human-96HE54	Human	United States	25
	GU391598	Violetti	*Canis lupus*	France	26
	FJ515309	Katze2	*Felis catus*	Switzerland	27
	CP000235	HZ	Human	United States	18
	GU236829	Dog-19	*Canis lupus fa*	Germany	25
	GU236808	human-96HE58	Human	United States	25

## Results

### Epidemiology

Between March 2009 and October 2010, information on 421 cases of fever of an unknown origin was collected from Peking University First Hospital, Laizhou People's Hospital in Shandong Province, 302 Military Hospital of the People's Republic of China, Yantai Infectious Diseases Hospital in Shandong Province, Beijing Friendship Hospital, and the Tianjin CDC. A total of 46 (10.9%) confirmed and 16 (3.8%) probable cases of anaplasmosis were diagnosed. The mean age of the investigated 62 confirmed and probable patients was 47 years (range: 18 to 78 years), and the ratio of men to women was 0.6. The age group with the highest incidence of anaplasmosis of the investigated 62 cases was the 50- to 59-year-old group (P<0.001). Of these patients, 25 were from Beijing, 4 were from Tianjian, 4 were from Hebei Province, 5 were from Henan Province, 16 were from Laizhou (Shandong Province) and 8 were from Yantai City (Shandong Province). Fifty-two patients (83.0%, 52/62) were farmers or individuals who worked in wild fields, and the family of each farmer patient was involved in raising domestic animals, many of which serve as hosts to ticks. The disease incidences occurred between March and October, with a peak during the tick breeding season between early March and late April. Overall, 48.4% (30/62) of the patients had been bitten by ticks, and eschars were observed on their bodies (Figures 1 F and G). A peak in the incidence of tick bites from April to July was noticed in the Shandong Peninsula, especially the Laizhou Bay area (Figure 1 B: area enclosed by the black circle), which is the largest wetland in the northern part of China and a famous post for migratory birds across Asia and the West Pacific (Figures 1 A, B and C).

### Laboratory diagnosis

#### Serological assay

To confirm that the febrile patients were infected with *A. phagocytophilum*, sera from the 421 patients were serologically tested according to the diagnostic criteria proposed by US CDC [1]. Five patients refused to provide blood samples during the convalescent phase of the illness, and another 15 patients could not be contacted to provide sera during the convalescent phase. The specific IgM and IgG antibodies against *A. phagocytophilum* were detected by IFA using the commercial FOCUS diagnostic reagents kit (REF: IF1403-A; Lot 102048, Cypress, CA, USA) according to the manufacturer's instructions. In 28 of the 42 (66.7%) sera samples obtained during the convalescent phase of the illness; there was a 4-fold increase in the IgG antibody titers, the highest of which were 1∶1280. Eleven confirmed cases and 4 probable cases were positive based on the IgM test of acute-stage sera. No samples were positive for the other ten rickettsial antigens in the differential diagnoses.

#### Molecular assay

The blood DNA extracted during the acute stage of illness was used for molecular diagnosis.

To enhance the sensitivity of the diagnosis, 3 types of assays were performed to detect DNA from the acute stage of illness. These assays included nested PCR, targeting the 16S rRNA gene; LAMP, targeting the *msp2* gene; and real-time PCR, targeting the *msp2* gene. For the *msp2* gene of *A. phagocytophilum*, 25 of the 62 (40.3%) patient DNA samples were positive according to LAMP, and two of these LAMP-positive samples were also confirmed by real-time PCR. Two DNA samples were positive based on the nested PCR and real-time PCR assays.

#### Morulae of *A. phagocytophilum*

**Figure 2:**
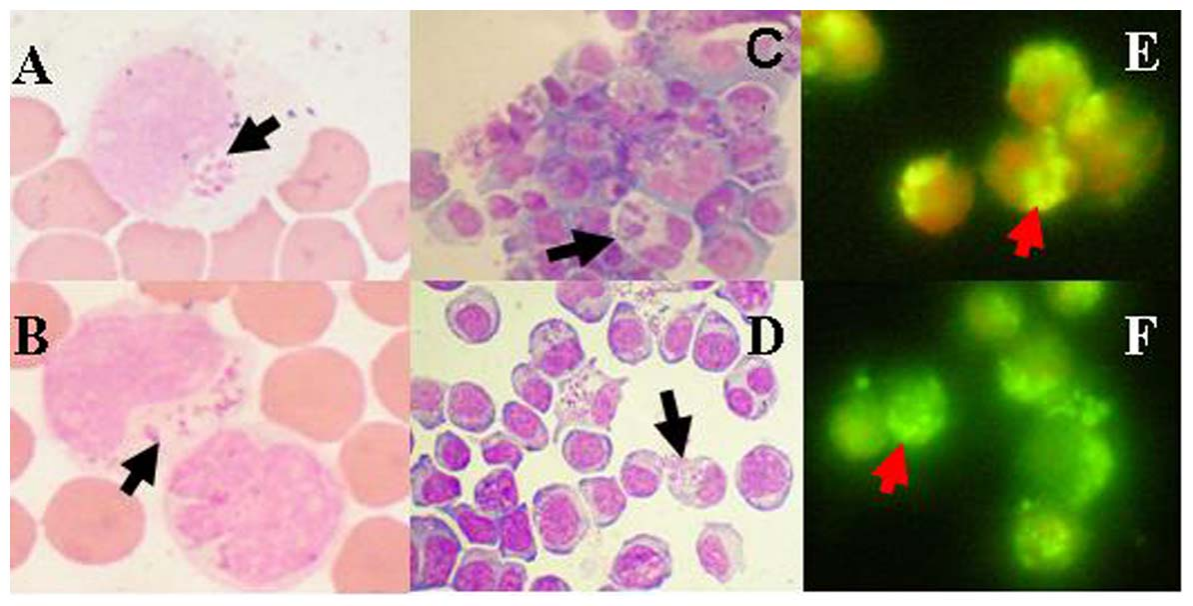
Morules in the neutrophils of HGA patients and morulae in the cultured HL60 cells. Morules(Wright's stain, mag×1000) in the neutrophils of the Patient15(A) and the Patient 37(B); Morulae (Wright's stain, mag×1000) in the cultured HL60 cells of the Patient 15(C) and the Patient 37(D) and morules(IFA,mag×600). in the cultured HL60 cells of the Patient 15(E) and the Patient 37(F).

The presence of morulae in neutrophils is a key indicator of *A. phagocytophilum* infection. Therefore, fresh buffy coat smears were prepared using the blood samples taken from patients during the acute stage of illness and stained with Wright's stain. The results of the morphological observation performed using a microscope showed that 6.5% (4/62) of the patients had typical morules in the neutrophils of their blood (Figure 2 A and 2B).

#### Isolation and identification of bacteria

To fulfill the major goal of the research, Chinese *A. phagocytophilum* bacteria were isolated by inoculating patients' fresh buffy coats into HL60 cells. The culture and isolate identification results indicated that patient 15 from Beijing, patient 26 from Hebei Province, and patients 37 and 46 from Shandong Province were positive based on Wright's stain and IFA at 7, 7, 10 and 12 days after inoculation, respectively. The cultures found to be positive based on Wright's stain (Patients 15 and 46) are shown in Figures 2 C and 2 D, and the positive results based on IFA(Patients 15 and 46) are shown in Figures 2 E and 2F. All four patients had been bitten by ticks prior to the onset of illness. The four human isolates were designated as BJ-HGA-agent-1, CZ-HGA-agent-2, LZ-HGA-agent-3 and LZ-HGA-agent-4. In addition, one tick isolate from the lymph blood of *H. longicornis* collected in Laizhou Bay in Shandong Province was obtained 12 days after inoculation, and the isolate was designated as LZ-HGA-agent-T1.

### Clinical features

The clinical manifestations of the 46 confirmed and 16 probable cases are summarized in Table 2. All patients were previously healthy. The median incubation was 7 days (range: 3 to 17 days), and 100% of the patients had a high fever, with a mean axillary temperature of 38.9°C (range: 38.0 to 41.5°C). In addition to fever, the interviewed patients reported the following common symptoms: chills (32.3%), headache (29.0%), malaise (87.1%), arthralgia (25.8%), and myalgia (29.0%). Typical and unique clinical features that are rarely reported from the United States and European countries included gastrointestinal complications (67.7% complained of anorexia, 35.5% of nausea, 29.0% of diarrhea, 19.4% of vomiting, and 21.0% of abdominal pain), slow pulse (24.2%) and facial edema (32.3%). hemorrhagic complications (18.9% complained of skin ecchymosis and 9.7% of melena) and mental confusion (16.1%) was observed in this study. A notable clinical feature of the Chinese anaplasmosis patients is that 26 (41.2%) of the patients were diagnosed with MODS, 5 (8.1%) of whom experienced rapidly worsening multiple organ failure and died. The main cause of death in these patients was the lack of treatment with anti-rickettsial medicine because of the misdiagnosis of other febrile illnesses in the patients' local village clinics during the acute stage of illness. Skin rash was present in 9.7% of patients, and 24.2% had local and regional lymph node enlargement. Most of the patients (90.3%) were empirically treated with antibiotics (doxycycline or tetracycline) specifically used to treat rickettsial diseases.

**Table 2:** Clinical and laboratory findings and laboratory diagnosis data in 46 confirmed and 16 probable cases in China, 2009–2010.

Clinical manifestations	Patients, n (%)	Laboratory findings	Patients, n (%)	Laboratory diagnosis	Patients, n (%)	Tick assays	Positive rates (%)
Eschars	30(48.4)	Leukopenia	61(98.4)	Morules in neutrophils	4(6.5)	Isolation of bacteria	4.0(1/25)^b^
Fever	62(100)	Thrombocyt-openia	56(90.3)	IgM positivity	15(24.2)	PCR amplification of the 16S rRNA gene	43.4(86/198)
Headache	18(29.0)	Elevated serum AST	62(100)	4-fold increase in IgG	28(66.7; 28/42)^a^		
Chills	20(32.3)	Elevated serum ALT	62(100)	Nested PCR	2(3.2)		
Weakness	54(87.1)	Elevated serum LDH	62(100)	Real-time PCR	4(6.5)		
Arthralgia	16(25.8)	Elevated serum CK	52(83.9)	LAMP	25(40.3)		
Myalgia	18(29.0)	Elevated serum BUN	45(72.6)	Isolation of bacteria	4(6.5)		
Anorexia	42(67.7)	Elevated serum creatinine	46(74.2)				
Nausea	22(35.5)	Elevated total bilirubin	52(83.9)				
Diarrhea	18(29.0)	Proteinuria (2+ to 4+)	50(80.6)				
Vomiting	12(19.4)	Hematuria (2+ to 4+)	23(37.1)				
Abdominal pain	13(21.0)	Elevated CRP	55(88.7)				
Skin rash	6(9.7)	Decreased HGB	27(40.3)				
Skin ecchymosis	8(12.9)	Extended APTT	32(51.6)				
Melena	6(9.7)	Elevated ESR	42(67.7)				
Lymph node enlargement	15(24.2)						
Slow pulse	15(24.2)						
Facial edema	20(32.3)						
Cough	15(24.2)						
Expiratory dyspnea	16(25.8)						
Jaundice	18(29.0)						
Mental confusion	10(16.1)						
Multiple organ dysfunction syndrome	26(41.2)						

AST, aspartate aminotransferase; ALT, alanine aminotransferase; LDH, lactate dehydrogenase; CK, creatine kinase; BUN, blood urea nitrogen; CRP, C-reactive protein; HGB, serum albumin; APTT, activated partial thromboplastin time; ESR, erythrocyte sedimentation rate.

a : Forty-two serum samples were obtained during the convalescent phase of the illness.

b : A total of 25 tick blood lymph sample pools were used to isolate the bacteria.

Because the 16 patients from Laizhou People's Hospital and the 8 patients from Yantai Infectious Diseases Hospital in Shandong Province were all hospitalized in ICU wards, an analysis of clinical manifestations of patients in different areas was conducted using the chi-square test. The results showed that the clinical manifestations of the patients from both hospitals were significantly more severe than those of patients from the other 3 areas. The incidence of patients with MODS and gastrointestinal and renal failure in these areas was significantly higher than in other areas (100% vs. 5.3%, P<0.0001). Similarly, the incidence of hemorrhagic complications was also higher in these areas than in the other 3 areas (75.0% vs. 10.5%, P<0.0001). The mortality rate in these areas was also higher than that in the other 3 areas (16.7% vs. 2.6%, P<0.05).

In addition to the general clinical manifestations, the abnormal laboratory markers in the 46 confirmed and 16 probable cases were analyzed and are also listed in Table 2. Low white blood cell counts (1.1–3.6×10^9^/L) were observed in 98.4% of the patients, and 90.3% of the patients had low platelet counts (10–102×10^9^/L); high levels of LDH (288–1700 U/L), CK (180–1202 U/L) and BUN (3.2–13.8 mmol/L); increased sedimentation rates (ESR, 21–90 mm/h); and increased hepatic transaminase levels, including AST(119–690 U/L) and ALT (145–513 U/L). In addition, 40.3% of the patients had low hemoglobin levels (32–152 g/L). Considering that 92.3% (24/26) of the patients with MODS were from the Laizhou Bay and Yantai areas of the Shandong Peninsula, the biochemical markers identified in patients from Laizhou Bay and the other 3 areas were compared and are summarized in Table 3. The results demonstrated that the WBC and platelet counts in the patients from Shandong Province were significantly lower than those in patients from other areas. However, some biochemical markers were significantly higher in the patients from Shandong Province than in patients from other areas.

**Table 3:** Compare of Laboratory markers between the patients in Shandong Laizhou Bay and the patients in other 3 areas in the study.

Laboratory marker	Patients in Shandong Laizhou Bay (n = 24)	Patients in other 3 areas (n = 38)	p-value
WBC count (×10^9^/L)	1.55±0.4	2.65±0.5	<0.001^a^
PLT count (×10^9^/L)	43.80±13.0	62.00±19.4	<0.0006
AST (U/L)	429.96±125.1	222.79±58.5	<0.001
ALT (U/L)	369.33±84.8	198.39±40.0	0.001
LDH (U/L)	1028.42±387.3	727.35±330.3	0.002
CK (U/L)	804.71±20.2	484.66±221.1	<0.001
BUN (mmol/L)	9.87±2.6	6.20±2.4	<0.001
APTT (s)	58.92±16.8	69.37±14.3	0.0132
HGB (g/L)	68.04±22.9	102.79±14.8	<0.001
ESR (mm/h)	69.79±12.3	40.75±12	<0.001

MODS, multiple organ dysfunction syndrome; SD, standard deviation; WBC, white blood cell; PLT, platelet; AST, aspartate aminotransferase; ALT, alanine aminotransferase; LDH, lactate dehydrogenase; CK, creatine kinase; BUN, blood urea nitrogen; PTT, activated partial thromboplastin time; HGB, hemoglobin; ESR, sedimentation rate.

a p<0.05, HGA patients without MODS vs HGA patients with MODS, according to student's t-test.

### Tick investigation and molecular assays

To determine the prevalence of *A. phagocytophilum* in ticks collected from the patients' domestic animals or wild fields, we retrospectively collected ticks from the patients' homes based on the history of tick bites. A total of 570 individual ticks were collected from Laizhou Bay, Shandong Province. Of these ticks, 114 were from wild fields and 456 were from dogs, cattle and goats. Ninety-five percent of the ticks were blood-engorged (Figure 1 E). The ticks were morphologically classified as *H. longicornis*, which is the dominant species in the central parts of China and is also distributed throughout the nation. DNA was extracted from 198 homogenized tick sample pools for nested PCR analysis targeting the 16S rRNA gene, and the results demonstrated that the prevalence of *A. phagocytophilum* in *H. longicornis* was 43.4% (86/198 sample pools).

### Sequence and phylogenetic tree analysis

#### 16S rRNA genes

**Figure 3:**
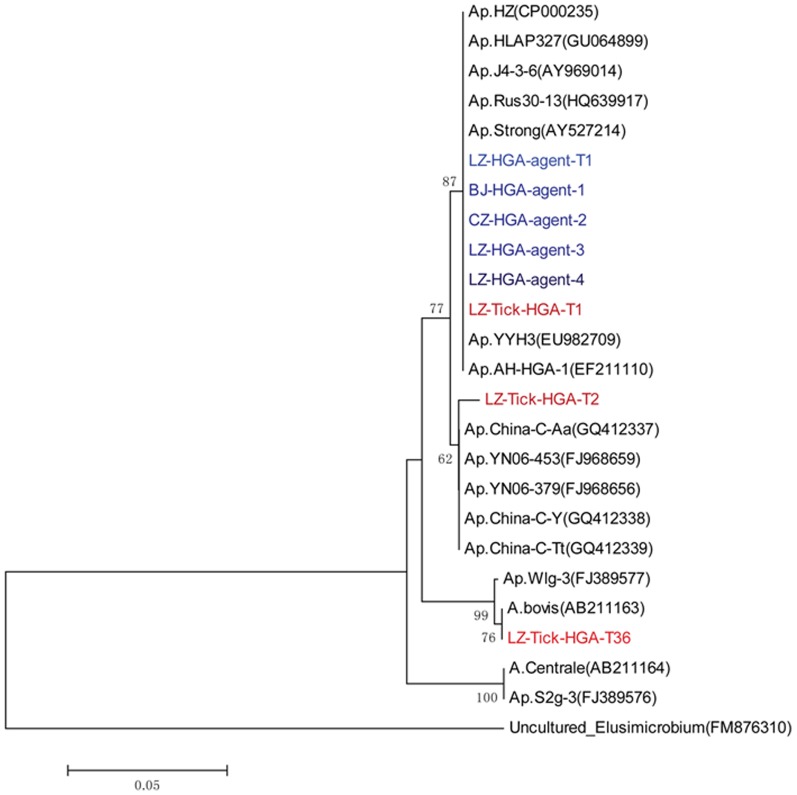
Phylogenetic tree based on the partial sequences of *A. phagocytophilum* 16S rRNA gene. Blue sequences: the five Chinese isolates of *A. phagocytophilum* identified in this study. Red sequences: *H. longicornis* isolates collected from the patients' domestic animals in Laizhou Bay, Shandong Province. Black sequences: sequences from patients, domestic animals, wild animals and ticks from other parts of the world. Green sequences:sequences from patient in Anhui Province in 2006 and patient in Yiyuan County, Shandong Province in 2007.

We performed phylogenetic analysis based on the partial sequences (725 bp) of the 16S rRNA genes to compare the sequences of the Chinese isolates of *A. phagocytophilum* with the sequences identified in ticks and in patients from previous studies in China and some sequences identified in patients, wild animals, domestic animals and ticks from other parts of the world (Figure 3). The results showed that the 16S rRNA gene sequences of the 5 isolates were 100% identical to each other and to the dominant tick sequence (LZ-Tick-HGA-T1), which accounted for up to 60% of the sequences identified in ticks from Laizhou, Shandong Province. A phylogenetic tree was then constructed. The results showed that there were 3 genetic groups of *A. phagocytophilum* represented in the study, and the 5 isolates and the above mentioned tick sequence (LZ-Tick-HGA-T1) were grouped into the same clan (100% identity) with the sequences (EF211110) that were identified during the nosocomial anaplasmosis transmission outbreak in Anhui Province in 2006 [4] and with the sequence (EU982709) of a patient from Yiyuan County, Shandong Province, in 2007 [24]; the sequence of the HLAP strain (GU064899) in *H. longicornis* from Jeju Island, Korea [19]; the sequence of the strain J4-3-6 (AY 969014) in *Ixodes ovatus* from Japan [21]; and the sequence of the strain Rus 30-13 (HQ 629917) in *Ixodes ricinus* from Russia [23]. In addition, we noticed that the 16S rRNA sequences of the five isolates were closely (99% identity) related to some Chinese isolates, including the strain China-C-Tt (GQ 412339) in *Tscherskia triton*, the strain China-C-Y (GQ412338) in domestic sheep, the strain China-C-Aa (GQ 412337) in *Apodemus agrarius* from the northeastern areas of China [20], the strain YN06-453 (FG968659) in *Niviventer niviventer* and the strain YN06-379 (FJ968656) in *Niviventer eha* from the southwestern areas of China.

#### *ank A* gene

It is known that the *ank A* gene is the critical component of *A. phagocytophilum*'s pathogenesis [28], [29], [30]. It is secreted by a type IV secretion system and functions by binding to DNA and altering the histone code to suppress or enhance gene transcription. This promotes resistance to killing, inflammatory recruitment of new host cells that can be infected, and delayed death of infected cells so that more are available to become infected from subsequent tick bites [31], [32], [33]. To assess the *ank A* gene sequences of the 5 Chinese isolates and further explore any potential relationship between the AnkA protein of the Chinese isolates and the severe clinical manifestations observed in China, we focused on amplifying and sequencing the *ank A* gene of the 5 Chinese isolates and compared the sequences with those of the *ank A* gene (from 725 to 2430 nt) of the strain HZ(CP000235). The results indicated the sequences of the human isolates from Beijing (Beijing-HGA-agent-1) and from Cangzhou City, Hebei Province, were 100% identical to each other but 97% identical to the strain HZ (variability in 53 bases). The sequences of 2 human isolates and the tick isolates (LZ-HGA-agent-3, LZ-HGA-agent-4 and LZ-HGA-agent-T1) from Shandong Province were 100% identical to each other but only 96.6% identical to that of the reference strain HZ (variability was noted in the 69 base).

**Figure 4:**
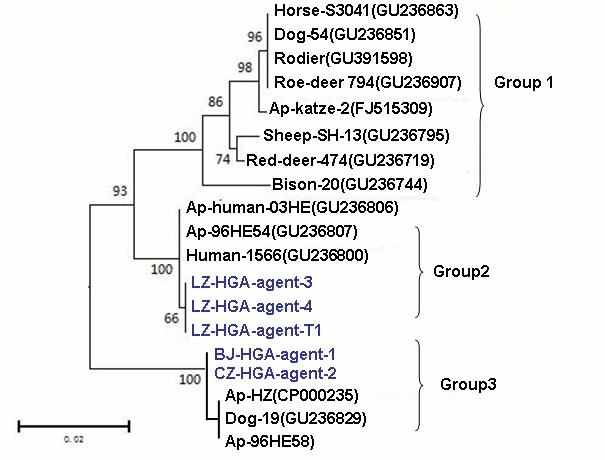
Phylogenetic tree based on the partial sequences of *A. phagocytophilum ank A* gene. Blue sequences:BJ-HGA-agent-1, CZ-HGA-agent-2, LZ-HGA-agent-3, LZ-HGA-agent-4, and LZ- HGA-agent-T1) and some *A. phagocytophilum* isolates identified in patients, domestic animals, wild animals and ticks from other parts of the world.

The phylogenetic analysis indicated that the 2 human isolates and the tick isolate from Shandong Province, which were associated with severe clinical manifestations, were independently grouped in the same clan (Figure 4), which was closely related to the strain human-03HE(GU236806) and the strain human-96HE54(GU236807) and human-1566(GU236800) from the United States [25]. The 2 isolates from Beijing and Cangzhou City were more closely related to the strain HZ. A interesting finding in the phylogenetic analysis is that the *ankA* gene sequences used in the study separated into two different gene clusters (Figure 4). The ruminant sequences were belonged to the group 1 while the human sequences were found in group 2 and group3 respectively. Similarity results were obtained by a recent study [25].

## Discussion

In China, the emerging tick-borne anaplasmosis disease was unrecognized in clinics until the first human infection with *A. phagocytophilum* observed in Anhui Province in 2006 [4], although the pathogen had been broadly identified in ticks, wild mice and domestic animals [3], [4], [5], [6], [7], [8], [9]. Since the first human case was identified, increasing numbers of clinicians have begun to recognize the zoonotic infectious disease. A total of 421 patients with fevers of unknown origin from the Provinces of Shandong, Hebei and Henan and the Cities of Beijing and Tianjing in China were admitted to the Peking University First Hospital, the Laizhou People's Hospital in Shandong Province, the 302 Military Hospital of the People's Republic of China, the Yantai Infectious Diseases Hospital in Shandong Province, the Beijing Friendship Hospital, and the Tianjin CDC from 2009 to 2010. Of these cases, 10.9% (46/421) were confirmed cases and 3.8% (16/421) were probable cases. Eighty-three percent of the patients were farmers, and 48.4% had been bitten by a tick. The peak incidence of this disease was from early March to late April. The mean susceptible age was 47 years (range: 18 to 78 years), and the ratio of men to women was 0.6. A significant finding of the study was that the Chinese cases featured severe clinical manifestations in addition to the general clinical manifestations that have been described in the United States and European countries. Among the Chinese patients, 41.2% had MODS; 22.6% had gastrointestinal complications; 12.9% had hemorrhagic complications; 24.2% had a slow pulse; 32.3% had facial edema; and 16.1% had mental confusion. These manifestations differed markedly from those reported from the United States and European countries [1], [2], [3]. Similarly, a high mortality rate of 8.1% was observed in this study, and the main cause of death was MODS. In addition to the general clinical manifestations, the laboratory test markers of the patients from Shandong Province (all patients with MODS) were strikingly different from those of the patients from other 3 areas (2 patients with MODS and 36 patients without MODS, who had significantly lower WBC counts and PLT counts but significantly elevated levels of LDH, CK, BUN, ALT and AST. These findings were similar to those documented in another recent clinical report in China [9]. In this study, we noticed that 92.3% (24/26) of the patients with MODS were from Laizhou Bay and Yantai, areas on the south shore of Bohai Bay (Figure 1B: area enclosed by the black circle). Bohai Bay is an important bird migration ‘transfer station’, wintering habitat and breeding haven in central Western Pacific and Northeast Asia (Figures 1 A, B and C).

Considering the above unique clinical findings of the patients with MODS and their specific geographical distribution in Shandong Province, the 16SrRNA gene and the *ankA* gene of the 2 human isolates and the tick isolates of *A. phagocytophilum* from this area were amplified and sequenced. The 16SrRNA gene is regarded as one of the most important candidate genes for exploring the genetic relationship among different strains of prokaryotic bacteria, while the *ankA* gene was deemed to be the key effecter of the pathogenesis of *A. phagocytophilum* infection. In addition, the *ankA* sequences of the 3 isolates from Shandong Province were also compared with those of 2 other isolates from Hebei Province and Beijing in the study, with some strains identified in the previous Chinese study and with some strains identified in ticks, wild animals, domestic animals and humans throughout the world.

In our analysis of the 16S rRNA gene, we found that the sequences of 16S rRNA were very conserved not only between the 2 human isolates and the tick isolates in Shandong Province but also between the other 2 isolates from Beijing and Cangzhou City, Hebei Province, in the study, as well as between the sequences identified in the patients from the outbreak of nosocomial transmission of anaplasmosis in Anhui Province in 2006 [4] and the sequence identified in the patient from Yiyuan County, Shandong Province, in 2007 [24].

Some recent reports from the United States have demonstrated that the *ankA* gene is the most important component in disease pathogenesis. This gene encodes a 153–160-kDa protein that differs according to geographic origin. As the major effector of HGA pathogenesis, *ankA* is secreted via the type IV secretion mechanism [33] through the cytoplasm and nuclear membrane to bind specific nuclear proteins. These results in transcriptional changes and a series of neutrophil functional alterations, promoting resistance to death and the inflammatory recruitment of new host cells that can be infected and delaying the death of infected cells so that more are available to be infected from subsequent tick bites [28], [34], [35]. Furthermore, a latest recent study reported that the diversity of *ankA* genes of *A.phagocytophilum* is related with the distinct host species [25], which might be determine their pathogenicity for heterologous hosts. In this situation, we specifically analyzed the *ankA* genes of the isolates in the study and performed a phylogenetic analysis. Unlike the sequences of the 16S rRNA genes, the sequences of the *ankA* genes of the isolates in our study had great variety. The sequences of the *ankA* genes of the 2 isolates and the tick isolates from Shandong Province were 100% identical to each other but significantly different from those of the 2 isolates from Beijing and Cangzhou (97.4% identical) and 97.0% identical to the strain HZ, from which the entire genome had been sequenced. Based on the phylogenetic analysis of the *ankA* gene, 3 groups were found based on the sequences used in the study. The 2 human isolates (LZ-HGA-agent-3 and LZ-HGA-agent-4) and the tick isolate (LZ- HGA-agent-T1) from Laizhou Bay, Shandong Province, where 100% patients had severe clinical features (MODS), were clustered in group 2 (Figure 4), which was closely with some US isolates (GU236806, GU236807 and GU236800). However, the other 2 human isolates from Beijing (BJ-HGA-agent-1) and Cangzhou City, Hebei Province (CZ-HGA-agent-2) in the study were clustered in group 3, which was more closely related with another US strain (strain HZ, CP000235). Most sequences from ruminant were found exclusively in group 1. Considering the diversity of *ankA* gene is involved in the pathogenicity of the agent such as host adaptation, environmental adaptation, functional diversification, modification of niche tropism, antigenic variation, and immune evasion, The molecular characterization of the *ankA* gene among these Chinese isolates might be meaningful. This genetic variation of the *ankA* gene among the isolates from Shandong Province may help us discern how the pathogen contributes to severe clinical manifestations in patients from these areas. However, the analysis in this study was limited due to the small sample sizes of culturable bacterial isolates from humans and ticks; therefore, additional data should be obtained in the future. Because most of the severely affected patients from Shandong Province had been bitten by ticks, a total of 570 *H. longicornis* isolates were collected from the patients' domestic animals and wild fields. Some research data from the United States and European countries have indicated that the *Ixodes* sp were the major vector of transmission of *A. Phagocytophilum* [36]. However, our study showed that *H. longicornis*, the dominant species of ticks throughout the mainland of China, was responsible for the disease. This finding is supported by a recent study in Hebei Province, where the prevalence of *A. phagocytophilum* in ticks was found to be 14.6% in *H. longicornis* and 30.8% in *D. nuttalli* [22].The prevalence of *A. phagocytophilum* in *H. longicornis* in Laizhou Bay, Shandong Province, was as high as 43.4% (86/198 sample pools). In this area, the dominant strain, represented by LZ-Tick-HGA-T1, accounted for up to 60% of the identified sequences in ticks [37]. The sequence of the 16S rRNA gene of LZ-Tick-HGA-T1 was 100% identical to the sequences of the 5 human isolates of *A. phagocytophilum* and the tick isolate (LZ-HGA-agent-T1) in this study; furthermore, it was 100% identical to the sequences identified in the patients from the nosocomial anaplasmosis transmission outbreak in Anhui Province in 2006 [4]; the sequence of the patient from Yiyuan County, Shandong Province, in 2007 [24]; and the sequences of *H. longicornis* from Jeju Island, Korea [19], *Ixodes ovatus* from Japan [17] and *Ixodes ricinus* from Russia. Briefly, this genetic group of *A. phagocytophilum* has a broad distribution throughout the Asian regions.

In conclusion, we successfully isolated the Chinese isolates of *A. phagocytophilum* and demonstrated the significant genetic diversity of the *ank A* genes of the Chinese isolates, which could help us to further research the relationship between variations in the *ank A* genes and the severity of the clinical manifestations observed in patients in China.
